# Dynamic and evolving response of pediatric medical emergencies in Rabat during the COVID-19 pandemic

**DOI:** 10.11604/pamj.supp.2020.35.2.24856

**Published:** 2020-07-16

**Authors:** Nour Mekaoui, Youssef Jeddi, Kenza Khachani, Badr sououd Benjelloun Dakhama, Lamya Karboubi

**Affiliations:** 1 Mohammed V University in Rabat, Morocco,; 2 Pediatric Medical Emergency Department of Rabat Morocco,; 3 Laboratory of Biostatistics, Clinical Research and Epidemiology, Faculty of Medicine and Pharmacy of Rabat Morocco

**Keywords:** Infectious diseases, paediatric emergency medicine, COVID-19

## To the Editors of the Pan African Medical Journal

The first case of COVID-19 was reported in China in December 2019 [1]. The WHO declared in March 11, 2020 the state of global pandemic. The first case in Morocco was declared in March 2nd, 2020. Morocco undertook containment measures from March 16, 2020 [2]. Restructuring measures of health structures leading to management of patients with COVID-19 have been taken while ensuring the protection of patients and caregivers [3]. Since the declaration of the first case, the children’s hospital in Rabat has established an evolving response plan for the healthcare sector, starting by providing information and training to the hospital staff about the disease, the preventive measures and the therapeutic modalities associated with the elaboration of pedagogic supports. The organization of the hospital circuit consisted of the establishment of a pre-triage unit at the entrance of the hospital where an emergency nurse equipped with PPE performed the sorting on the careful interrogation, which determined the suspected cases depending on the definition and the measure of the temperature. The increase in the number of cases lead the hospital set up three separate circuits with marking on the ground: A COVID circuit (red) for confirmed COVID-19 patients, with a direct way to the dedicated hospital service.

A suspect COVID circuit (blue) intended to welcome all patients with Acute Respiratory Insufficiency was set up in consultation rooms outside the emergency department including a doctor, a nurse with individual protection measures, an equipped consultation room with a waiting room with respect for distancing measures. In case of a suspicious case, the patient is referred to the isolation service for nasopharyngeal swab and management. A clean or classic circuit (green): after elimination of the suspected cases according to epidemiological and clinical criteria, the patient is then sent home or to hospitalization in a non-COVID service. From March 2 to May 30, 314 children consulted at the “suspect COVID” consultation. The median age was 5 years with extremes ranging from 12 days to 16 years. There was a discreet male dominance with a ratio sex M/F of 1.9. The patients came mainly from their home (95.8%), during the day (70%) and came on their own (92%). After a clinical examination and careful interrogation, 65 cases (21.25%) met the current definition of suspected cases and presented contact with a positive subject and or presented respiratory and/or digestive signs without obvious diagnosis. Only one patient was tested positive after PCR diagnosis. 60.6% were treated at home, 8.4% were hospitalized in isolation, 30.6% in general pediatrics and only one patient were hospitalized in intensive care. All of these measures have enabled us to optimize the management of suspected corona virus cases while protecting the patients consulting for other urgent diseases.
